# Blending an internet-based emotion regulation intervention with face-to-face psychotherapy: Findings from a pilot randomized controlled trial

**DOI:** 10.1016/j.invent.2023.100650

**Published:** 2023-07-20

**Authors:** Laura Luisa Bielinski, Tobias Krieger, Marijke Amanda Kley, Franz Moggi, Thomas Berger

**Affiliations:** a Department of Clinical Psychology and Psychotherapy, University of Bern, Bern, Switzerland; b University Hospital of Psychiatry and Psychotherapy, University of Bern, Bern, Switzerland

**Keywords:** Blended psychotherapy, Emotion regulation, Transdiagnostic

## Abstract

**Background:**

Transdiagnostic interventions targeting shared mechanisms may improve treatment of mental health disorders. One way of providing such interventions is through blended treatment. This study examined the addition of an internet-based emotion regulation intervention to face-to-face psychotherapy in an outpatient setting.

**Methods:**

In a pilot randomized controlled trial, 70 patients with a range of diagnoses were assigned to an internet-based program targeting emotion regulation + treatment as usual (face-to-face psychotherapy; TAU) (*n* = 35) or TAU (*n* = 35). Assessments occurred at baseline, after six, and after 12 weeks and included measures of symptom severity, emotion regulation, and various intervention feasibility parameters.

**Results:**

ITT-analyses revealed no significant group-by-time interaction for the primary and almost all secondary outcomes. Descriptively, between-group effect sizes were in favor of the intervention group for almost all outcomes. Sensitivity analysis with patients who completed a minimum of three modules of the internet-based program showed a significant group-by-time interaction for the Difficulties in Emotion Regulation Scale in favor of the intervention group. The internet-based intervention showed good satisfaction ratings, user experience and usability. Findings from therapist measures complemented patient measures.

**Conclusion:**

Preliminary results show that an internet-based emotion regulation intervention added to psychotherapy may not reduce symptom severity compared to psychotherapy alone. The intervention was rated positively by patients and therapists regarding several parameters, but certain features still need to be improved. An RCT powered to detect small between-group effect-sizes is necessary to consolidate findings.

## Highlights

• The blending of an internet-based emotion regulation intervention with face-to-face psychotherapy (TAU) was examined
• Findings suggest that the internet-based intervention added to TAU may not reduce symptom severity compared to TAU
• The internet-based intervention had good patient satisfaction and usability ratings
• An improved version of the intervention should be examined within a larger RCT

## 1 Introduction

Treatments for mental health disorders are often applied to treat one specific diagnosis. However, not all patients in diagnosis-led treatments achieve clinical recovery and often patients do not suffer from just one mental health disorder but from a range of problems (Dalgleish et al., 2020). This concern with comorbidity and heterogeneity of clinical presentations (Dalgleish et al., 2020), along with the effort involved for therapists to master several disorder-specific protocols (Sauer-Zavala et al., 2017), make transdiagnostic interventions an appealing option (Newby et al., 2015).

Different definitions of transdiagnostic interventions or treatments exist. For example, McEvoy et al. (2009, p. 21) describe these types of treatments as “those that apply the same underlying treatment principles across mental disorders without tailoring the protocol to specific diagnoses”. Dalgleish et al. (2020) distinguish between “hard” transdiagnostic approaches that may replace the diagnostic system with alternative frames of reference and “soft” approaches that maintain the underlying diagnostic classification while aiming to focus on processes or develop interventions that pertain to one or more diagnoses. Sauer-Zavala et al. (2017) build on previous definitions of transdiagnostic processes (Mansell et al., 2009; Harvey et al., 2011) and distinguish between the following categories of transdiagnostic treatments: universally applied therapeutic principles, modular treatments and treatments targeting shared mechanisms across classes of disorders. They describe the targeting of shared mechanisms as potentially having benefits beyond the other two. Several such shared mechanisms have been proposed (Harvey et al., 2004, Harvey et al., 2011).

One potential transdiagnostic construct that has received increased attention in recent years is emotion regulation (ER). Gross (1998) describes ER as the processes by which we influence which emotions we have, when we have them, and how we experience and express them. The extended process model (EPM, Gross, 2015) describes different phases of ER: identification, selection, and implementation. Problems at these different phases may lead to symptoms of psychopathology (Gross, 2015). ER may be pertinent to the development and treatment of psychopathology (Aldao et al., 2010; Berking and Wupperman, 2012; Lincoln et al., 2022). In recent years, psychotherapeutic treatment approaches have placed more specific emphasis on ER. Examples include Affect Regulation Training (ART; Berking et al., 2013) and Emotion Regulation Therapy (Mennin and Fresco, 2014). A more detailed list of approaches and interventions with an emphasis on ER can be found in a previous publication (Bielinski et al., 2020). Some of these interventions may be useful supplements to therapy with the goal of overall improved treatment, as has been shown, for example, for a shortened version of ART (Berking et al., 2013).

A new way of delivering an ER intervention as an add-on to psychotherapy is by integrating technology. Combining an internet-based intervention and face-to-face psychotherapy is termed blended treatment (BT, Bielinski et al., 2021). Research on BT has made strides in recent years and studies point to efficacy and effectiveness (Thase et al., 2018; Nakao et al., 2018). While most blended research is disorder-specific (Berger et al., 2018; Kooistra et al., 2016) studies on transdiagnostic BT are ongoing (Schaeuffele et al., 2022; Osma et al., 2021). Recently, an internet-based ER-training was examined as an add-on to psychotherapy for adolescents (Wisman et al., 2023). The authors showed that compared to psychotherapy alone, the ER-training as an add-on to psychotherapy was superior regarding a reduction in depressive and anxiety symptoms as well as maladaptive ER and regarding an increase of adaptive ER at the six months follow-up timepoint (Wisman et al., 2023).

The aim of this study was to examine an internet-based ER intervention based on the EPM as an add-on to face-to-face psychotherapy for outpatient adults with a range of diagnoses. Thus, we compared an intervention group receiving outpatient psychotherapy + access to REMOTION to a control group receiving face-to-face psychotherapy only (TAU) regarding a broad set of outcome measures. An essential goal of the study, in addition to the assessment of possible effects of REMOTION, was to evaluate the added value of REMOTION in routine outpatient psychotherapy treatment with indicators such as intervention usage and user experience and with assessments from the patient and therapist perspective.

## 2 Method

The full study protocol has been published (Bielinski et al., 2020) and is summarized below.

### 2.1 Trial design

An intervention group with access to REMOTION during routine outpatient psychotherapy was compared to TAU that received psychotherapy only (TAU). Assessments took place at baseline (T0), after 6 weeks (T1) and after 12 weeks (T2). The TAU group received access to REMOTION after T2. The Ethics Committee of the Canton of Bern approved the study (2019-01929), and the trial was registered with ClinicalTrials.gov (NCT04262726). The study aimed to recruit 70 patients randomly allocated 1:1 to each condition.

### 2.2 Participants

**Fig. 1:**
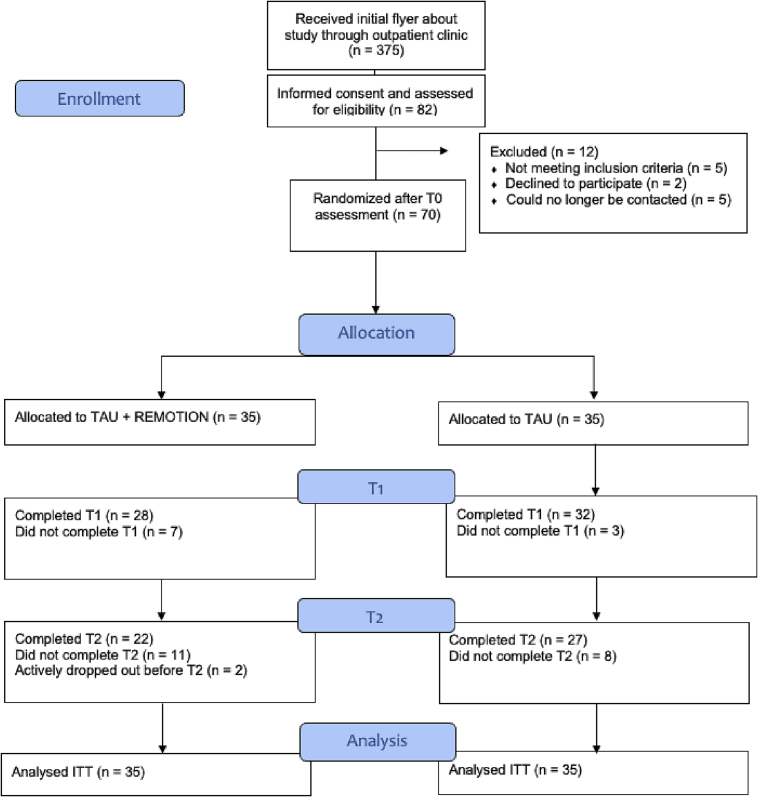
Patient-flow.

Recruitment took place at the outpatient clinic of University of Bern between February 2020 and August 2022. Patients learned about the study at first contact with the clinic. Patients were included if they provided informed consent (IC) and fulfilled the following: a) age over 18 years b) being in psychotherapeutic treatment at the outpatient clinic (initiated treatment at the clinic) c) fulfilling criteria of a mental illness (*DSM-IV*, Sass et al., 1996), and d) having internet access. Exclusion criteria included a) current participation in another intervention specifically for ER b) current episode or a history of psychotic disorders or bipolar disorder, c) acute suicidality, and d) not being fluent in German language. Seventy patients were randomized (Fig. 1).

### 2.3 Therapists

Once a patient agreed to participate, we asked their therapist about participation. Honoring natural routine-care conditions, therapists were not randomly assigned to patients. Patients were treated by a total of 36 therapists. Thirty therapists (83.3 %) were female. The mean age of therapists was 35.31 years (*SD* = 6.59). Therapists completed training in integrative Cognitive Behavioral Therapy (CBT) at the University of Bern or were still in said training during the study period. On average, therapists treated 1.94 (*SD* = 1.31, range = 1–6) study patients. Thirteen therapists (36.1 %) treated patients in both conditions.

### 2.4 REMOTION + TAU

**Fig. 2:**
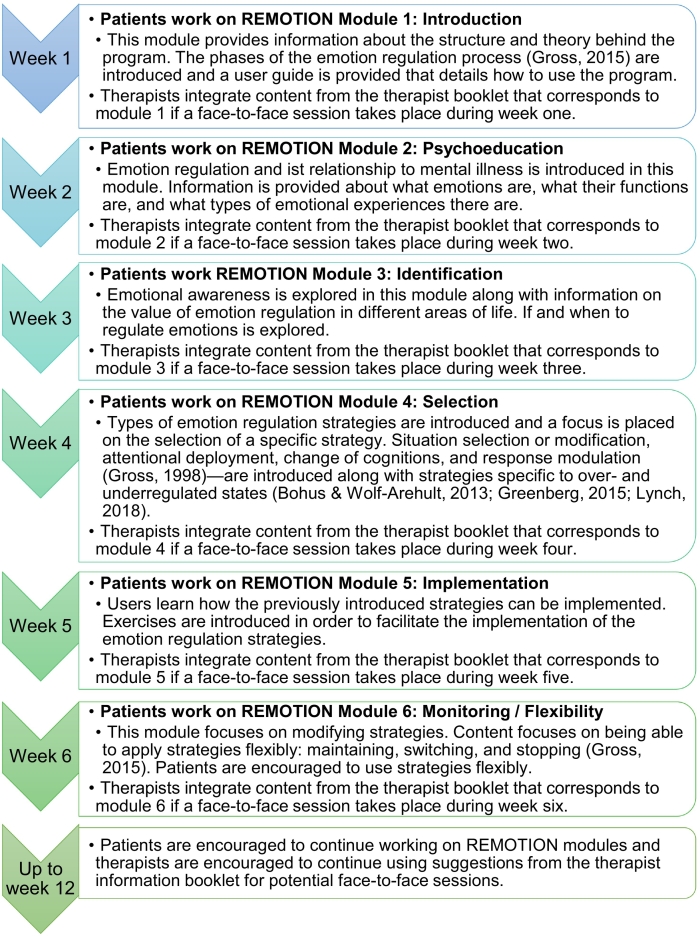
The suggested timing and structure of the blended treatment and the content for each module of the web-based program REMOTION are depicted. Note that the description of REMOTION content is adapted from a figure in a previous publication (Bielinski et al., 2020).

The intervention group received access free of charge to REMOTION as an add-on to psychotherapy. REMOTION is a six-module internet-based intervention (IBI) developed by LLB and TB with input from FM. The general structure is based on the EPM (Gross, 2015). Within the intervention, content from different treatment approaches is integrated (see Bielinski et al., 2020 for a complete overview). For example, the intervention includes content for over and underregulated states (Bohus and Wolf-Arehult, 2013; Greenberg, 2015; Lynch et al., 2015) REMOTION is a web-based program that includes text, video and audio material. One module of REMOTION takes approximately 30–120 min to complete. Patients were instructed to work on one module a week immediately after randomization up to T1 and patients were asked to continue using the intervention to T2 (see Fig. 2). The research team sent patients weekly standardized reminders to work on one module a week up to T1 and one more reminder to continue working on the program content at week 9. Therapists received an intervention information booklet immediately after patient randomization with general information on the web-based program and with suggestions on how to integrate the program into face-to-face sessions. They were informed that patients ideally worked on one module a week and content in the therapist information booklet was designed to complement each module of the REMOTION intervention. Honoring routine conditions, the timing and number of face-to-face sessions was not preset in the study and could vary for each patient. Further details on the intervention content and on therapist information booklet content can be found in a previous publication (Bielinski et al., 2020).

### 2.5 TAU

TAU consisted of integrative CBT provided at the University of Bern outpatient clinic. Therapy at the clinic focuses on CBT principles and places a focus on case conceptualization based on Consistency Theory (Grawe, 1998), Plan Analysis (Caspar, 2018) and Motive-oriented Therapeutic Relationship (Caspar, 2019).

### 2.6 Procedure

Patients who had received information on the REMOTION study at their first contact with the outpatient clinic, who fulfilled all inclusion and none of the exclusion criteria, were randomized into one of two conditions after baseline assessment. The allocation list was produced using an automated computer-generated random numbers table and concealed from investigators and therapists. After randomization, patients and therapists were informed about randomization outcome. Participants in the intervention group received an access code for REMOTION and information on program use immediately after randomization. They were informed to work on one module of REMOTION per week for six weeks and then to continue using the intervention after the six week timepoint. Therapists of patients in the intervention group were given the REMOTION therapist information booklet immediately after randomization outcome was communicated. At T1 and T2 participants were contacted via email and asked to complete the questionnaires. Participants who did not complete the questionnaires, received three reminders.

### 2.7 Measures

A detailed description of all measures used can be found in the study protocol (Bielinski et al., 2020). Questionnaires were administered over the internet.

#### 2.7.1 Primary outcome

General symptom severity was measured with the Brief Symptom Inventory (BSI, German version; Franke, 2000), containing 53 items. For the analyses in this study, the Global Severity Index (BSI-GSI) is reported. Cronbach's alpha in the current sample was 0.95 (T0).

#### 2.7.2 Secondary outcome measures and other measures

##### 2.7.2.1 Emotion regulation

Difficulties in ER were assessed with the Difficulties in Emotion Regulation Scale (DERS; Gratz and Roemer, 2004; German version; Ehring et al., 2008). The DERS is a 36-item self-report-questionnaire used to assess patient difficulties in ER and comprises six subscales. Items are filled out on a five-point scale ranging from 1 = *almost never* to 5 = *almost always*. Cronbach's alpha in the current sample was 0.93 (T0). Emotion competencies/skills were assessed with the *Fragebogen zur standardisierten Selbsteinschätzung emotionaler Kompetenzen* (SEK-27) questionnaire, a 27-item self-report instrument and comprises nine subscales (Berking and Znoj, 2008). The SEK-27 instructions ask individuals to fill out items with regard to the timeperiod of the past week. Cronbach's alpha in the current sample was 0.95 (T0).

##### 2.7.2.2 Depressive and anxiety symptoms

Depressive symptoms were assessed with the Patient Health Questionnaire-9 (PHQ-9; Löwe et al., 2002). Cronbach's alpha in the current sample was 0.82 (T0). Anxiety symptoms were assessed with the Generalized Anxiety Disorder Scale-7 (GAD-7, German version; Löwe et al., 2008). Cronbach's alpha in the current sample was 0.82 (T0).

##### 2.7.2.3 Health-related quality of life and well-being

Health-related quality of life was assessed with the Short Form Health Survey-12 (SF-12, Ware et al., 1996). This is a frequently used questionnaire (Gandek et al., 1998) that examines physical and psychological aspects. Ware et al. (1996) report acceptable to good test–retest reliability for both aspects. Patient well-being was assessed with the World Health Organization-Five Well-Being Index (WHO-5; German version; Brähler et al., 2007). Cronbach's alpha in the current sample was 0.80 (T0).

##### 2.7.2.4 Self-compassion

The Self-Compassion Scale (SCS; Neff, 2003; German version Hupfeld and Ruffieux, 2011) is a 26-item self-report scale that is reliable and valid (Hupfeld and Ruffieux, 2011). Cronbach's alpha in the current sample was 0.91 (T0).

##### 2.7.2.5 Working alliance

The Working Alliance Inventory—short revised (WAI-SR, Wilmers et al., 2008) is a 12-item self-report scale with good psychometric properties. Cronbach's alpha in the current sample was 0.88 (T1). The WAI-SR was given to patients to fill out at T1 and T2.

##### 2.7.2.6 Intervention feasibility parameters

An overview of measures recorded for the REMOTION + TAU group specifically concerning the internet-based intervention, can be found in Table 1. Additionally, semi-structured interviews were conducted of which results have been published (Bielinski et al., 2022).

**Table 1:** Internet-based intervention feasibility parameters (REMOTION + TAU group only).

Construct	Measure
Adherence/usage of the web-based program	Number of pages visited
	Number of modules completed
	Number of exercises completed
	Time spent in the intervention
Usability	System Usability Scale (SUS; Brooke, 1996)
User-experience	MeCUE-questionnaire (version 2.0, Minge, 2018; Minge and Riedel, 2013)
Satisfaction	Client Satisfaction Questionnaire (CSQ-8; Schmidt et al., 1989; adapted for internet interventions)
Negative effects	Inventory to Assess Negative Effects of Psychotherapy (INEP; Ladwig et al., 2014, adapted for internet interventions)

##### 2.7.2.7 Therapist measures

In the REMOTION + TAU condition only, therapists' perceived effect of REMOTION on therapy was assessed. One quantitative item recorded helpfulness (*did you perceive REMOTION as helpful for therapeutic work in face-to-face psychotherapy with this patient on a scale of 1 (not helpful) to 5* (*helpful*)). This was complemented by four open-ended questions (Supplementary Material B). The DERS (German version; Ehring et al., 2008) and the SEK-27 (Berking and Znoj, 2008) were adapted and given to therapists at the same measurement timepoints as patients. The therapists were asked to fill out the same questions as the patients but from an observer perspective. The wording of the questions was changed as little as possible from the originals. Cronbach's alpha was for both the modified DERS and SEK-27 (labeled DERS_T_ and SEK-27_T_) 0.92 at T0.

#### 2.7.3 Contamination between conditions

Due to the within-therapist design, possible contamination between REMOTION + TAU and TAU was controlled in the following ways: number of therapists who provided therapies in both conditions was recorded and therapists who provided both were asked not to talk about REMOTION content or use the exercises provided in the therapist booklet during a TAU therapy, a strategy utilized previously (Magill et al., 2019). Adherence to these constraints was recorded by asking therapists if they used the specific therapist booklet exercises during their TAU therapies at each assessment timepoint (one item with a yes/no answer format for each therapist booklet exercise along with one item on the frequency of use of said element).

### 2.8 Sample size and power considerations

Due to the pilot nature of the study and there being no previous studies on the effects of internet-based ER interventions as add-ons to psychotherapy at the time of study conceptualization, no a priori power analysis was conducted. However, sample size recommendations for pilot studies to inform RCTs were considered (Sim and Lewis, 2012; Whitehead et al., 2016).

### 2.9 Statistical analyses

To analyze group differences for demographic data and primary and secondary outcomes at T0, *t*-tests (or non-parametric alternatives) and χ^2^-tests or Fisher's exact tests were conducted. The primary and secondary outcomes were analyzed on an intention-to-treat (ITT) basis using mixed-model repeated-measures analysis of variance with time (T0-T1-T2) as within-group factor and treatment as between-group factor. With this approach, missing values do not need to be imputed, but parameters of the values are estimated (Gueorguieva and Krystal, 2004). Separate models were estimated for each outcome. The mixed models were estimated using a Restricted Maximum Likelihood approach. The covariance structure with best fit according to Bayesian Information Criterion was selected for each model. Within- and between-group effect sizes (Cohen's *d*) were calculated based on estimated means and pooled standard deviations from the observed means. In a sensitivity analysis, data from patients in the intervention group who completed a minimum of three modules (*n* = 25) was compared to data from the TAU group (*n* = 35) using mixed-model repeated-measures analysis of variance. Group differences on the WAI-SR were assessed with Mann-Whitney *U* tests (non-normally distributed data). Parameters concerning the IBI (REMOTION + TAU group only) were analyzed descriptively. Therapist measures on perceived impact of REMOTION on therapy were analyzed descriptively for the quantitative item and qualitatively for four open-ended questions (Braun and Clarke, 2022, Supplementary Material B). Therapist-rated ER parameters were analyzed using mixed-model repeated-measures analysis of variance using the same steps outlined above. Therapist items on contamination between conditions were analyzed descriptively.

## 3 Results

### 3.1 Baseline patient sample descriptives

There were no between-group differences for demographic characteristics at baseline (Table 2). There were no baseline between-group differences on any of the primary or secondary outcome measures (*p*s > .16). As part of routine clinical practice, diagnosis was assessed with a Structured Clinical Interview for DSM-IV (SCID; Wittchen et al., 1997) conducted by a therapist or therapist in training at the outpatient clinic, or clinical judgment if no SCID-interview was conducted.

**Table 2:** Patient sample at baseline.

	Total (*N* = 70)	Intervention group (*n* = 35)	TAU (*n* = 35)	Statistic
Mean age (*SD*), *Mdn*, range	31.29 (11.02), 27.50, 18–63	30.26 (9.72), 27.00, 18–58	32.31 (12.25), 28.00, 18–63	*U* = 578.50, *p* = .69
Female, *n* (%)	50 (71.4)	28 (80.0)	22 (62.9)	*Χ*^*2*^_*(1)*_ = 2.52, *p* = .19
Marital status, *n* (%)				Fisher's Exact Test, *p* = .18
Single	48 (68.6)	27 (77.1)	21 (60.0)	
In partnership/married	19 (27.1)	6 (17.1)	13 (37.1)	
Separated/divorced	3 (4.3)	2 (5.7)	1 (2.9)	
Education, *n* (%)				Fisher's Exact Test, *p* = .93
Compulsory school	1 (1.4)	1 (2.9)	0 (0)	
Apprenticeship without/with vocational diploma	10 (14.3)	5 (14.3)	5 (14.3)	
High school diploma	14 (20.0)	8 (22.9)	6 (17.1)	
University/university of applied sciences	42 (60.0)	20 (57.1)	22 (62.9)	
Other	3 (4.3)	1 (2.9)	2 (5.7)	
Employment, *n* (%)				Fisher's Exact Test, *p* = .16
Full-time	18 (25.7)	5 (14.3)	13 (37.1)	
Part-time	20 (28.6)	13 (37.1)	7 (20.0)	
Unemployed	7 (10.0)	3 (8.6)	4 (11.4)	
Student	23 (32.9)	13 (37.1)	10 (28.6)	
No answer	2 (2.9)	1 (2.9)	1 (2.9)	
Nationality, *n* (%)				Fisher's Exact Test, *p* = .75
Swiss	61 (87.1)	30 (85.7)	31 (88.6)	
German	5 (7.1)	2 (5.7)	3 (8.6)	
Other	4 (5.7)	3 (8.6)	1 (2.9)	
Primary diagnosis				Fisher's Exact Test, *p* = .38
Mood disorders (depressive disorders)	29 (41.4)	11 (31.4)	18 (51.4)	
Anxiety disorders	10 (14.3)	7 (20.0)	3 (8.6)	
Adjustment disorders	12 (17.1)	6 (17.1)	6 (17.1)	
Somatoform disorders	6 (8.6)	4 (11.4)	2 (5.7)	
Eating disorders	7 (10.0)	5 (14.3)	2 (5.7)	
Substance-related disorders	5 (7.1)	2 (5.7)	3 (8.6)	
Personality disorders	1 (1.4)	–	1 (2.9)	
With comorbid diagnoses, *n* (%)	29 (41.4)	14 (40)	15 (42.9)	*Χ*^*2*^_*(1)*_ = 0.06, *p* = 1.00
Number of comorbid diagnoses for patients with more than one diagnosis, *n* (%)				
One	21 (72.4)	10 (71.4)	11 (73.3)	
Two	6 (20.7)	2 (14.3)	4 (26.7)	
Three or more	2 (6.90)	2 (14.3)	0 (0)	
Currently taking psychopharmaceuticals, *n* (%)	17 (24.3)	11 (31.4)	6 (17.1)	*Χ*^*2*^_*(1)*_ = 1.94, *p* = 0.27
Past psychological treatment, *n* (%)	40 (57.1)	20 (57.1)	20 (57.1)	*Χ*^*2*^_*(1)*_ = 0.00, *p* = 1.00

### 3.2 Drop-out, face-to-face sessions, and control of contamination

Sixty patients (85.7 %) completed T1 assessment and 49 (70.0 %) T2 (Fig. 1). An assessment was considered complete if the primary outcome questionnaire was completed. There were no statistically significant differences at baseline on most demographic and outcome measures between completers and T2 non-completers (*ps* > .17). However, non-completers had lower symptom severity (BSI-GSI) than completers (*U* = 293.50, *p* = .01), lower depressive symptoms (PHQ-9) than completers (*U* = 346.50, *p* = .03) and higher ER skills (SEK-27) than completers (*t* = −2.10, *p* = .04). The average number of face-to-face sessions from randomization to T1 was 4.51 (*SD* = 2.32) in the intervention group (*n* = 35) and 4.46 (*SD* = 2.42) in TAU (n = 35). The average number of face-to-face sessions from randomization to T2 was 8.12 (*SD* = 3.42) in the intervention group (*n* = 33) and 7.74 (*SD* = 3.32) in TAU (*n* = 35). Group differences regarding number of face-to-face sessions were not statistically significant at both timepoints (*ps* > .65). Since some therapists provided both conditions, we asked if elements suggested in the therapist booklet were applied during TAU therapies. This was the case for 6 TAU therapies. Details on use of elements from the therapist booklet in these 6 TAU therapies can be found in Supplementary Material B.

### 3.3 Effects for primary and secondary outcomes (ITT)

Observed and estimated means are presented in Table 3. For the BSI-GSI, the group-by-time interaction effect was not statistically significant, *F*(2,108.64) = 0.83, *p* = .44. Between-group effect sizes favored the intervention group and were at *d* = 0.13 at T1, and at *d* = 0.37 at T2. Regarding secondary outcomes, almost all group-by-time interaction effects were not significant (*ps* > .26), and effect sizes were in favor of the intervention group. A significant group-by-time interaction effect was found for the SCS, *F*(2,103.81) = 3.55, *p* = .03, with a small between-group effect size of *d* = −0.26 in favor of the intervention group at T1 that was close to zero at T2 (*d* = 0.06).

**Table 3:** Observed and estimated means and within- and between-group effect sizes (ITT sample).

Measure	Baseline		T1 (observed)		T1 (estimated)		T2 (observed)		T2 (estimated)		Group-by-time interaction	T0 to T1 within-group effect sizes (estimated means)	T0 to T2 within-group effect sizes (estimated means)	Between group effect sizes at T1 (estimated means)	Between group effect sizes at T2 (estimated means)
	Mean (SD)	n	Mean (SD)	n	Mean (SE)	n	Mean (SD)	n	Mean (SE)	n	F, df	Cohen's d (95 % CI)	Cohen's d (95 % CI)	Cohen's d (95 % CI)	Cohen's d (95 % CI)
BSI-GSI															
Intervention	0.97 (0.55)	35	0.79 (0.45)	28	0.79 (0.09)	35	0.73 (0.46)	22	0.68 (0.10)	35	F_2,108.64_ = 0.83, *p* = .44	0.36 (−0.11 to 0.83)	0.57 (0.09 to 1.05)	0.13 (−0.34 to 0.60)	0.37 (−0.11 to 0.84)
TAU	1.00 (0.49)	35	0.83 (0.49)	32	0.85 (0.09)	35	0.92 (0.52)	27	0.86 (0.09)	35		0.31 (−0.17 to 0.78)	0.28 (−0.19 to 0.75)		

DERS															
Intervention	101.80 (23.95)	35	89.93 (19.99)	28	90.69 (4.01)	35	90.26 (20.21)	21	90.90 (4.55)	35	F_2,109.35_ = 1.35, *p* = .26	0.50 (0.03 to 0.98)	0.49 (0.02 to 0.97)	0.41 (−0.06 to 0.88)	0.28 (−0.19 to 0.75)
TAU	102.03 (19.11)	35	98.34 (22.24)	32	99.36 (3.84)	35	97.96 (26.15)	27	97.45 (4.11)	35		0.13 (−0.34 to 0.60)	0.20 (−0.27 to 0.67)		

SEK-27															
Intervention	59.54 (20.59)	35	68.68 (13.80)	28	68.60 (3.09)	35	63.81 (17.57)	21	65.84 (3.35)	35	F_2,106.36_ = 0.12, *p* = .88	−0.52 (−0.99 to −0.04)	−0.33 (−0.80 to 0.14)	−0.24 (−0.71 to 0.23)	−0.12 (−0.58 to 0.35)
TAU	57.66 (15.57)	35	65.47 (15.88)	32	65.06 (2.98)	35	62.44 (17.39)	27	63.83 (3.11)	35		−0.47 (−0.95 to 0.00)	−0.37 (−0.85 to 0.10)		

GAD-7															
Intervention	8.03 (4.53)	35	6.82 (4.05)	28	6.74 (0.80)	35	7.10 (4.73)	21	6.84 (0.88)	35	F_2,109.68_ = 0.25 *p* = .78	0.30 (−0.17 to 0.77)	0.26 (−0.21 to 0.73)	0.30 (−0.17 to 0.77)	0.20 (−0.27 to 0.67)
TAU	8.57 (3.78)	35	7.84 (4.61)	32	8.04 (0.76)	35	7.96 (4.51)	27	7.76 (0.81)	35		0.13 (−0.34 to 0.60)	0.20 (−0.28 to 0.66)		

PHQ-9															
Intervention	10.23 (5.75)	35	8.50 (5.50)	28	8.50 (0.96)	35	7.76 (5.76)	21	7.47 (1.08)	35	F_2,108.97_ = 1.08, *p* = .34	0.31 (−0.16 to 0.78)	0.48 (0.00 to 0.96)	0.27 (−0.20 to 0.74)	0.46 (−0.02 to 0.93)
TAU	10.43 (4.55)	35	9.59 (4.54)	32	9.88 (0.93)	35	10.33 (4.99)	27	9.93 (0.99)	35		0.12 (−0.35 to 0.59)	0.11 (−0.36 to 0.57)		

WHO-5															
Intervention	10.20 (4.48)	35	11.50 (4.45)	28	11.29 (0.85)	35	12.23 (4.87)	21	12.29 (0.97)	35	F_2,112.25_ = 0.33, *p* = .72	−0.24 (−0.71 to 0.23)	−0.45 (−0.92 to 0.03)	−0.23 (−0.70 to 0.24)	−0.43 (−0.90 to 0.05)
TAU	8.77 (4.32)	35	10.28 (5.14)	32	10.20 (0.81)	35	10.00 (5.05)	27	10.16 (0.87)	35		−0.30 (−0.77 to 0.17)	−0.30 (−0.77 to 0.18)		

SF-12_PH_															
Intervention	52.99 (7.41)	35	53.36 (6.77)	28	53.17 (1.52)	35	52.81 (6.79)	21	52.10 (1.64)	35	F_2,108.89_ = 0.27, *p* = .76	−0.03 (−0.49 to 0.44)	0.13 (−0.34 to 0.59)	−0.39 (−0.86 to 0.09)	−0.25 (−0.72 to 0.22)
TAU	50.43 (9.61)	35	49.69 (10.50)	32	49.73 (1.47)	35	49.31 (9.64)	26	50.04 (1.55)	35		0.07 (−0.40 to 0.54)	0.04 (−0.43 to 0.51)		

SF-12_MH_															
Intervention	34.66 (7.73)	35	37.00 (6.95)	28	36.89 (1.50)	35	38.02 (7.11)	21	38.15 (1.71)	35	F_2,111.42_ = 0.17, *p* = .84	−0.30 (−0.77 to 0.17)	−0.47 (−0.95 to 0.01)	−0.25 (−0.72 to 0.22)	−0.40 (−0.88 to 0.07)
TAU	32.59 (7.92)	35	35.06 (10.02)	32	34.76 (1.44)	35	35.27 (9.56)	26	34.75 (1.57)	35		−0.24 (−0.71 to 0.23)	−0.25 (−0.72 to 0.22)		

SCS															
Intervention	2.61 (0.68)	35	2.91 (0.63)	28	2.93 (0.11)	35	2.84 (0.66)	21	2.82 (0.12)	35	F_2,103.81_ = 3.55, *p* = .03	−0.49 (−0.96 to −0.01)	−0.31 (−0.79 to 0.16)	−0.26 (−0.73 to 0.21)	0.06 (−0.41 to 0.53)
TAU	2.73 (0.53)	35	2.79 (0.51)	32	2.78 (0.11)	35	2.86 (0.65)	26	2.86 (0.11)	35		−0.10 (−0.57 to 0.37)	−0.22 (−0.69 to 0.25)		

*Note*. BSI = Brief Symptom Inventory (Franke, 2000), DERS = Difficulties in Emotion Regulation Scale (Ehring et al., 2008). SEK-27 = Fragebogen zur standardisierten Selbsteinschätzung emotionaler Kompetenzen (Berking and Znoj, 2008), GAD-7 = Generalized Anxiety Disorder Scale-7 (Löwe et al., 2008). PHQ-9 = Patient Health Questionnaire-9 (Löwe et al., 2002). WHO-5 = WHO-Five Well-Being Index (Brähler et al., 2007). SF-12_MH_: Short Form Health Survey mental health subscale; SF-12_PH_: Short Form Health Survey physical health subscale (Gandek et al., 1998; Ware et al., 1996). SCS = Self-Compassion Scale (Hupfeld and Ruffieux, 2011).

### 3.4 Effects for the sample that completed three or more internet-based modules

Analyses with the intervention group sample that completed a minimum of three modules (*n* = 25) are presented in Supplementary Material A. This sample was compared with the full TAU group. For the BSI-GSI, the group-by-time interaction effect was not statistically significant, *F*(2,97.07) = 1.24, *p* = .30. Between-group effect sizes were in favor of the intervention group (*d* = 0.13 at T1, *d* = 0.30 at T2). Regarding the secondary outcome measures, the group-by-time interaction effect was significant for the DERS, *F*(2,98.28) = 3.35, *p* = .04, with a medium between-group effect size in favor of the intervention group at T1, *d* = 0.51, and a small effect size in favor of the intervention group at T2, *d* = 0.35. The group-by-time interaction effect was significant for the SCS, *F*(2,96.40) = 6.94, *p* = .002, with a small between-group effect size of *d* = −0.26 in favor of the intervention group at T1 and no effect (*d* = 0.07) at T2. For all other outcomes no significant group-by-time interaction was found (ps > .15).

### 3.5 Working alliance

For patients who completed the WAI-SR, Mann-Whitney *U* tests showed no statistically significant differences between the two groups at T1 (*U* = 308.50, *p* = .06) or at T2 (*U* = 266.50, *p* = .89). Between-group effect sizes were in favor of TAU at T1 (*d* = 0.48), with no effect at T2 (*d* = 0.08).

### 3.6 The internet-based intervention

#### 3.6.1 Adherence and usage

**Fig. 3:**
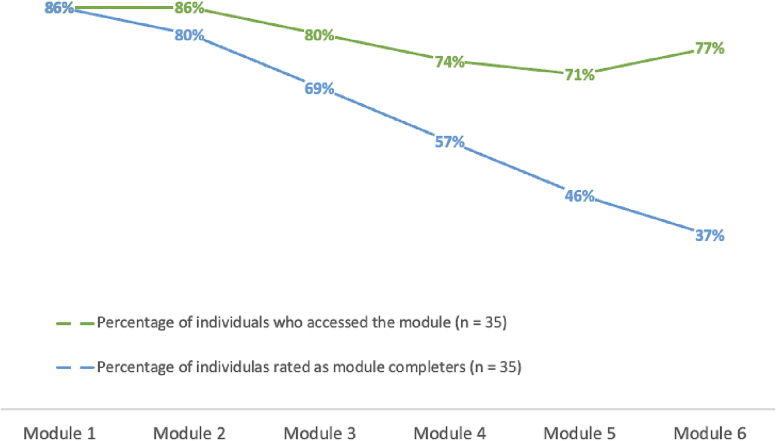
Access and completion rates per module at T2.

REMOTION consists of six modules with 62 main pages and 10 main exercises with possibility for user-input. Thirty out of 35 patients (85.7 %) randomized to the intervention group logged on to REMOTION. At T1, the average number of different pages visited for the 35 individuals was 39.09 (*SD* = 22.67). The average number of modules completed was 3.40 (*SD* = 2.03). A module was considered completed if every page of the module was visited. The average number of exercises completed was 4.43 (*SD* = 3.48). The average time patients spent within the program up to T1 was 154 min (*SD* = 146 min). In these computations, periods of inactivity of 15 min or longer were subtracted. From randomization to T2 for all 35 individuals, the average number of different pages visited was 41.17 (*SD* = 23.22). The average number of modules completed was 3.74 (*SD* = 2.21), the average number of exercises completed 4.54 (*SD* = 3.56). Module completion rates for the 35 individuals are shown in Fig. 3. The average time participants spent in the program up to T2 was 179.52 min (*SD* = 185.12).

#### 3.6.2 System usability and user experience

The average SUS score was 79.4 (*SD = 13.80, T1, n = 27*). Values above 68 are considered good usability (Brooke, 1996). Twenty-six patients in the REMOTION group filled out the MeCUE-questionnaire 2.0 item measuring experience of the product as a whole at T1 (*M* = 2.45, *SD* = 2.20). A higher score can be interpreted as better (scale −5 = as bad to 5 = as good).

**Fig. 4:**
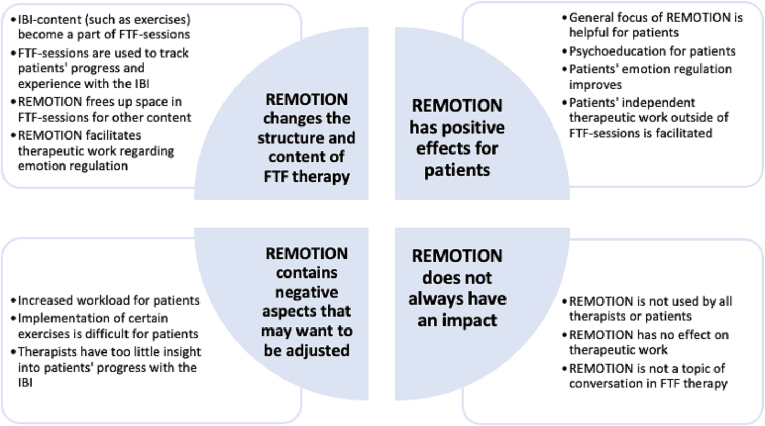
Thematic analysis of therapists' perceived impact of REMOTION on therapy.

#### 3.6.3 Satisfaction and negative effects

The average CSQ-8 score for the internet-based program at T1 was 2.95 (*SD* = 0.58, *n* = 27). An average score of 2.95 corresponds most closely to being *mostly satisfied* with the program (1 = *quite dissatisfied*, 2 = *mildly dissatisfied*, 3 = *mostly satisfied*, 4 = *very satisfied*). A more detailed analysis of patient satisfaction on the item level can be found in Supplementary Material A. One patient who completed the INEP-questionnaire reported a negative effect at T1. This individual felt slightly more burdened by past life events due to REMOTION.

### 3.7 Therapist measures

REMOTION was rated as being *rather helpful* for therapeutic work in face-to-face psychotherapy (M = 3.88, *SD* = 0.90, *n* = 24, T1) on the quantitative item. For the open-ended questions, four main themes were generated (Fig. 4, Supplementary Material B). Observed and estimated means for therapist ratings of patient emotion regulation are presented in Supplementary Material B. For the DERS_T_ the group-by-time interaction was not statistically significant, *F*(2, 96.65) = 1.40, *p* = .25. This was also the case for the SEK-27_T_, *F*(2, 95.75) = 0.86, *p* = .43.

## 4 Discussion

An intervention group that received access to an internet-based ER intervention as an add-on to psychotherapy was compared to TAU. ITT analyses indicated no significant group-by-time interaction effect for the primary and almost all secondary outcomes. Descriptively, effect sizes were in favor of the intervention group for almost all outcomes. These findings can be compared to a study on an internet-based ER intervention added to CBT for adolescents (Wisman et al., 2023). The authors found effects in favor of the intervention group on symptomatology and ER at 6 months. However, they examined depressive and anxiety disorders only, TAU was disorder-specific CBT, program usage was complemented by separate face-to-face/screen-to-screen contacts in addition to psychotherapy and effects were found at follow-up. Perhaps, a longer assessment period may have been necessary to identify intervention effects in our study. Moreover, TAU in our study consisted of integrative CBT. This may have made it harder to find effects in favor of the intervention group but may also be representative of routine conditions.

Our sensitivity analysis indicated that for an intervention like REMOTION to be effective at impacting emotion regulation, it may be particularly relevant to ensure patients receive sufficient exposure to the intervention. This supports the argument that IBIs as add-ons to face-to-face psychotherapy may be more effective than psychotherapy alone when an increased intervention dose is provided (Berger et al., 2018). Notably, overall time spent with the internet-based program was higher in the study by Berger et al. (2018) than in the current study, however the program examined by Berger et al. (2018) also included more modules. Suggestions that are potentially relevant to improving intervention usage are provided in a previous publication (Bielinski et al., 2022) and include better integration of internet-based and face-to-face elements and giving therapists access to patient intervention progress.

REMOTION showed good patient satisfaction, usability, and user experience. There were no significant differences between intervention group and TAU regarding the patient-rated working alliance, descriptively effect sizes were in favor of TAU. Some possible explanations that may help explain this finding are discussed in a previous publication (Bielinski et al., 2022). For example, patient interviews mentioned that patient knowledge gain from the intervention may have been problematic for the therapist and and that therapists had too little knowledge of the internet-based intervention. Therapist interviews described an increased workload for patients negatively impacting the therapeutic relationship. Previous studies examining the working alliance in blended vs. face-to-face therapies have shown working alliance ratings to be comparable between the two treatment formats (Askjer and Mathiasen, 2021; Ly et al., 2015).

Regarding study feasibility, of the 375 individuals who received initial study information per post, 82 provided IC. While this may not necessarily be due to the internet intervention itself (for example, perhaps in some cases the study was deemed too effortful), it does call into question the reach of the intervention and perhaps also the dissemination strategy. Research shows that disseminating internet interventions through the same modality as the intervention is likely to minimize behavioral gaps between being offered an intervention and engaging with it (Batterham et al., 2022). Moreover, patients who did not complete assessment measures during the study differed with regard to reported symptom severity at baseline compared to assessment completers, alluding to the possibility that patients may be less motivated for study participation if symptomatology is less severe.

Finally, analyses of therapist measures complemented patient measures. There were no significant differences between the intervention group and TAU regarding ER parameters as rated by therapists. Therapists rated REMOTION as helpful for face-to-face therapy and qualitative analyses revealed that therapists felt REMOTION incurred benefits for session structure and patients. Negative aspects were also noted. For example, therapists felt they did not have enough access to the intervention and certain exercises were deemed unhelpful. Such elements would need to be modified prior to a larger RCT. This RCT should then be powered to detect small between-group effect sizes.

### 4.1 Strengths and limitations

This study has several strengths. It is the first to examine an internet-based ER intervention based on the EPM, REMOTION, as an add-on to face-to-face psychotherapy for outpatient adults. Several measures were assessed from both the patient's and therapist's perspectives and Structured Clinical Interviews for DSM-IV (Wittchen et al., 1997) were conducted during the study. Finally, we used an active control group (integrative-CBT) that reflects routine conditions. This can also be seen as a limitation making it harder to find between-group effects. Also, the patient sample was mainly female and highly educated, thus limiting the generalizability of the results. ER was assessed with global questionnaires and that therapists' versions of the ER questionnaires have not been validated. A further limitation concerns the duration of the recruitment period, during which contextual variables may have changed and the lack of follow-up assessment in the study. Finally, due to the fact that sample size was small, any results regarding effectiveness remain preliminary only.

## 5 Conclusion

This pilot RCT provides preliminary evidence that an internet-based ER intervention added to psychotherapy may not reduce symptom severity in comparison to psychotherapy only but may reduce ER difficulties for patients that completed 3 or more modules of the internet-based intervention. The intervention was deemed useful by patients and therapists; patient satisfaction, usability and user experience were rated positively. In a next step, an improved version of the intervention should be examined in a larger RCT.

The following are the supplementary data related to this article.

Supplementary Material A Results from the analyses regarding patient satisfaction and from the sensitivity analyses. Supplementary Material A

Supplementary material B Analyses of the therapist measures. Supplementary material B

## Declaration of competing interest

Research was funded by the Department of Clinical Psychology and Psychotherapy, 10.13039/100009068University of Bern, Switzerland. The authors declare that they have no known competing financial interests or personal relationships that could have appeared to influence the work reported.
