# Supplementary material for: Blending an internet-based emotion regulation intervention with face-to-face psychotherapy: Findings from a pilot randomized controlled trial
Source: Internet Interv. 2023 Jul 20;33:100650. doi: 10.1016/j.invent.2023.100650 (PMC10413058; doi:10.1016/j.invent.2023.100650)
Supplement: Supplementary Material A — Results from the analyses regarding patient satisfaction and from the sensitivity analyses. [file mmc1.pdf]

Supplementary Material A

1. **Analysis of patient satisfaction (CSQ-8, German version, Schmid et al. 1989, adapted for internet interventions) on the item level at T1**

A more detailed analysis of patient satisfaction on the item level at T1 showed that 16 patients (59.3%) rated the quality of the program as *good*, 9 patients (33.3%) rated the quality as *excellent,* and 2 patients (7.4%) rated it as *fair*. With regard to the program helping patients deal more effectively with their problems, a total of 16 patients (59.3%) answered *yes, it helped somewhat*, 5 patients (18.5%) answered *yes, it helped a great deal*, and the remaining 6 patients (22.2%) answered *no, it really did not help*. With regards to overall satisfaction, 16 patients (59.3%) said that they were *mostly satisfied*, 5 patients (18.5%) were *very satisfied*, 5 patients (18.5%) considered themselves *mildly dissatisfied*, and one patient (3.7%) was *quite dissatisfied*.

2. **Mixed-model repeated-measures analysis of variance with the sample that completed a minimum of three modules of REMOTION**

The following table provides details on the analyses with the intervention group sample that completed three or more modules (n = 25) compared to TAU (n = 35). Results for the primary and secondary outcomes are displayed. Estimated and observed means, the group-by-time interaction and effect sizes at both T1 and T2 are displayed. There were no baseline differences between the two groups for the primary and all secondary outcome measures (ps > .12).

Table A1. Observed and estimated means and within and between-group effect sizes for the intervention group sample that completed three or more modules (*n* = 25) and TAU (*n* = 35)

	**T0 (baseline)**		**T1 (observed)**		**T1 (estimated)**		**T2 (observed)**		**T2 (estimated)**		**Group-by-time-Interaction**	**T1 within-group effect sizes (estimated means)**	**T2 within-group effect sizes (estimated means)**	**Between group effect sizes at T1 (estimated means)**	**Between group effect sizes at T2 (estimated means)**
Measure	Mean (SD)	n	Mean (SD)	n	Mean (SE)	n	Mean (SD)	n	Mean (SE)	n	F, df	Cohens *d (95% CI)*	Cohens *d (95% CI)*	Cohens *d (95% CI)*	Cohens *d (95% CI)*
**BSI-GSI**															
Treatment	1.09 (0.58)	25	0.77 (0.44)	23	0.80 (0.10)	25	0.71 (0.47)	18	0.73 (0.11)	25	$F_{2,97.07}$ = 1.24 *p*	0.56 (−0.00 to 1.13)	0.68 (0.11 to 1.25)	0.13	0.30 (−0.22 to 0.82)
Control	1.00 (0.49)	35	0.83 (0.49)	32	0.86 (0.09)	35	0.92 (0.52)	27	0.88 (0.09)	35	= .30	0.29 (−0.19 to 0.76)	0.24 (−0.23 to 0.71)	(−0.39 to 0.64)	
**DERS**															
Treatment	104.92 (24.32)	25	87.09 (20.02)	23	88.40 (4.55)	25	88.03 (20.96)	18	89.06 (4.98)	25	$F_{2,98.28}$ = 3.35,	0.74 (0.17 to 1.32)	0.70 (0.13 to 1.27)	0.51	0.35
Control	102.03 (19.11)	35	98.34 (22.24)	32	99.38 (3.86)	35	97.96 (26.15)	27	97.43 (4.12)	35	p = .04	0.13 (−0.34 to 0.60)	0.20 (−0.27 to 0.67)	(−0.01 to 1.04)	(−0.17 to 0.86)
**SEK-27**															
Treatment	56.64 (20.72)	25	69.78 (14.50)	23	68.69 (3.49)	25	64.39 (18.81)	18	65.14 (3.72)	25	$F_{2,98.\ 2}$ = 0.77 *p*	−0.67 (−1.24 to −0.10)	−0.43 (−0.99 to 0.13)	−0.24	−0.07
Control	57.66 (15.57)	35	65.47 (15.88)	32	65.06 (2.96)	35	62.44 (17.39)	27	63.83 (3.09)	35	= .47	−0.47 (−0.95 to 0.00)	−0.37 (−0.85 to 0.10)	(−0.75 to 0.28)	(−0.59 to 0.44)
**GAD-7**															
Treatment	8.60 (4.71)	25	6.26 (3.60)	23	6.48 (0.90)	25	6.67 (4.70)	18	6.86 (0.98)	25	$F_{2,98.51}$ = 0.94 p	0.51 (−0.06 to 1.07)	0.37 (−0.19 to 0.93)	0.37	0.20 (−0.32 to 0.71)
Control	8.57 (3.78)	35	7.84 (4.61)	32	8.03 (0.76)	35	7.96 (4.51)	27	7.77 (0.81)	35	= .39	0.13 (−0.34 to 0.60)	0.19 (−0.28 to 0.66)	(−0.15 to 0.89)	
**PHQ-9**															
Treatment	10.64 (6.20)	25	7.78 (4.98)	23	8.27 (1.08)	25	7.00 (5.35)	18	6.94 (1.18)	25	$F_{2,97.92}$ = 1.96,	0.42 (−0.14 to 0.98)	0.64 (0.07 to 1.21)	0.34	0.53 (0.01 to 1.05)
Control	10.43 (4.55)	35	9.59 (4.54)	32	9.88 (0.92)	35	10.33 (5.86)	27	9.94 (0.98)	35	p = .15	0.12 (−0.35 to 0.59)	0.09 (−0.38 to 0.56)	(−0.18 to 0.86)	
**WHO-5**															
Treatment	10.28 (4.51)	25	12.00 (4.22)	23	11.65 (0.96)	25	12.67 (4.90)	18	12.59 (1.06)	25	$F_{2,101.\ 5}$ = 0.27,	−0.31 (−0.87 to 0.24)	−0.49 (−1.05 to 0.07)	−0.30	−0.49 (−1.01 to
Control	8.77 (4.31)	35	10.28 (5.14)	32	10.20 (0.81)	35	10.00 (5.05)	27	10.15 (0.87)	35	*p* = .76	0.30 (−0.77 to 0.17)	−0.29 (−0.77 to 0.18)	(−0.82 to 0.21)	0.03)
**SF-12$_{MH}$**															
Treatment	34.15 (7.61)	25	37.31 (6.22)	23	37.06 (1.69)	25	39.34 (6.75)	18	38.82 (1.87)	25	$F_{2,100.91}$ = 0.40,	−0.42 (−0.98 to 0.14)	−0.65 (−1.22 to −0.08)	−0.27	−0.48 (−1.0 to
Control	32.59 (7.92)	35	35.06 (10.02)	32	34.77 (1.43)	35	35.27 (9.54)	26	34.78 (1.57)	35	*p* = .67	−0.24 (−0.71 to 0.23)	−0.25 (−0.72 to 0.22)	(−0.78 to 0.25)	0.05)
**SF-12$_{PH}$**											$F_{2,99.13}$ = 0.30,			−0.38	
Treatment	52.74 (7.57)	25	53.32 (7.28)	23	53.26 (1.79)	25	52.54 (7.11)	18	52.17 (1.90)	25	*p* = .75	−0.07 (−0.63 to 0.49)	0.08 (−0.48 to 0.63)	(−0.90 to 0.14)	−0.25 (−0.76 to
Control	50.43 (9.61)	35	49.69 (10.50)	32	49.73 (1.52)	35	49.31 (9.64)	26	50.03 (1.59)	35		0.07 (−0.40 to 0.54)	0.04 (−0.43 to 0.51)		0.27)
**SCS**															
Treatment	2.49 (0.64)	25	2.97 (0.67)	23	2.93 (0.12)	25	2.85 (0.71)	18	2.81 (0.13)	25	$F_{2,96.\ 0}$ = 6.94,	−0.67 (−1.24 to −0.10)	−0.47 (−1.04 to 0.09)	−0.26	0.07 (−0.44 to 0.59)
Control	2.73 (0.53)	35	2.79 (0.51)	32	2.78 (0.11)	35	2.86 (0.65)	26	2.86 (0.11)	35	*p* = .002	−0.10 (−0.57 to 0.37)	−0.22 (−0.69 to 0.25)	(−0.77 to 0.26)	

Note. BSI = Brief Symptom Inventory (Franke, 2000), DERS = Difficulties in Emotion Regulation Scale (Ehring et al., 2008). SEK-27 = Fragebogen zur standardisierten Selbsteinschätzung emotionaler Kompetenzen (Berking & Znoj, 2008), GAD-7 = Generalized Anxiety Disorder Scale-7 (Löwe et al., 2008). PHQ-9 = Patient Health Questionnaire-9 (Löwe et al., 2002). WHO-5 = WHO-Five Well-Being Index (Brähler et al., 2007). SF-12$_{MH}$: Short Form Health Survey mental health subscale; SF-12$_{PH}$: Short Form Health Survey physical health subscale (Gandek et al., 1998; Ware et al., 1996). SCS = Self-Compassion Scale (Hupfeld & Ruffieux, 2011).

References in Supplementary Material A:

Berking, M., & Znoj, H. (2008). Development and validation of a self-report measure for the assessment of emotion regulation skills (SEK-27). *Zeitschrift für Psychiatrie, Psychologie und Psychotherapie, 56*(2), 141–153. https://doi.org/10.1024/1661-4747.56.2.141

Brähler, E., Mühlan, H., Albani, C., & Schmidt, S. (2007). Teststatistische Prüfung und Normierung der deutschen Versionen des EUROHIS-QOL Lebensqualität-Index und des WHO-5 Wohlbefindens-Index. *Diagnostica, 53*(2), 83–96. https://doi.org/10.1026/0012-1924.53.2.83

Ehring, T., Fischer, S., Schnülle, J., Bösterling, A., & Tuschen-Caffier, B. (2008). Characteristics of emotion regulation in recovered depressed versus never depressed individuals. *Personality and Individual Differences, 44*(7), 1574–1584. https://doi.org/10.1016/j.paid.2008.01.013

Franke, G. H. (2000). *BSI. Brief Symptom Inventory–Deutsche Version. Manual.* Beltz.

Gandek, B., Ware, J. E., Aaronson, N. K., Apolone, G., Bjorner, J. B., Brazier, J. E., Bullinger, M., Kaasa, S., Leplege, A., Prieto, L., & Sullivan, M. (1998). Cross-validation of item selection and scoring for the SF-12 Health Survey in nine countries: Results from the IQOLA Project. *Journal of Clinical Epidemiology, 51*(11), 1171–1178. https://doi.org/10.1016/S0895-4356(98)00109-7

Hupfeld, J., & Ruffieux, N. (2011). Validation of a German version of the Self-Compassion Scale (SCS-D). *Zeitschrift für Klinische Psychologie und Psychotherapie: Forschung und Praxis, 40*(2), 115–123. https://doi.org/10.1026/1616-3443/a000088

Löwe, B., Decker, O., Müller, S., Brähler, E., Schellberg, D., Herzog, W., & Herzberg, P. Y. (2008). Validation and standardization of the Generalized Anxiety Disorder Screener (GAD-7) in the general population. *Medical Care, 46*(3), 266–274. https://doi.org/10.1097/MLR.0b013e318160d093

Löwe, B., Spitzer, R. L., Zipfel, S., & Herzog, W. (2002). *Gesundheitsfragebogen für Patienten (PHQ-D). Manual und Testunterlagen.* (2. Aufl.). Medizinische Universitätsklinik Heidelberg. Karlsruhe: Pfizer. https://www.klinikum.uni-heidelberg.de/fileadmin/Psychosomatische_Klinik/download/PHQ_Manual1.pdf

Schmidt, J., Lamprecht, F., & Wittmann, W. W. (1989). Satisfaction with inpatient care: Development of a questionnaire and first validity assessments. *PPmP: Psychotherapie Psychosomatik Medizinische Psychologie, 39*(7), 248–255.

Ware, J. E., Kosinski, M., & Keller, S. D. (1996). A 12-Item Short-Form Health Survey: construction of scales and preliminary tests of reliability and validity. *Medical Care*, 220-233. http://dx.doi.org/10.1097/00005650-199603000-00003
