# Supplementary material for: Blending an internet-based emotion regulation intervention with face-to-face psychotherapy: Findings from a pilot randomized controlled trial
Source: Internet Interv. 2023 Jul 20;33:100650. doi: 10.1016/j.invent.2023.100650 (PMC10413058; doi:10.1016/j.invent.2023.100650)
Supplement: Supplementary material B — Analyses of the therapist measures. [file mmc2.pdf]

Supplementary material B: Analyses of the therapist measures

1. Contamination of TAU

Use of exercises from the REMOTION therapist booklet in TAU face-to-face sessions was reported by therapists for 6 TAU therapies. Table B1 shows a detailed depiction of contamination for these 6 therapies, as recorded at post-assessment and follow-up assessment timepoints. The item assessing how often a specific REMOTION therapist material exercise was used had the following answer format: *never, rarely, sometimes, often, mostly*.

**TAU Therapy**	**REMOTION therapist material exercise used that corresponds to REMOTION module 1**		**REMOTION therapist material exercise used that corresponds to REMOTION module 2**		**REMOTION therapist material exercise used that corresponds to REMOTION module 3**		**REMOTION therapist material exercise used that corresponds to REMOTION module 4**		**REMOTION therapist material exercise used that corresponds to REMOTION module 5 (underregulated states)**		**REMOTION therapist material exercise used that corresponds to REMOTION module 5 (overregulated states)**	
	Baseline to post-assessment timepoint	Post to follow-up assessment timepoint	Baseline to post-assessment timepoint	Post to follow-up assessment timepoint	Baseline to post-assessment timepoint	Post to follow-up assessment timepoint	Baseline to post-assessment timepoint	Post to follow-up assessment timepoint	Baseline to post-assessment timepoint	Post to follow-up assessment timepoint	Baseline to post-assessment timepoint	Post to follow-up assessment timepoint
**1**	Sometimes	Often	-	Sometimes	-	Often	Sometimes	Sometimes	-	-	Sometimes	Often
**2**	Often	-	-	Often	-	-	Sometimes		Often	Often	-	-
**3**	-	-	-	-	-	-	Sometimes	Sometimes	-	-	-	Sometimes
**4**	-	-	-	Sometimes	-	-	-	-	-	-	-	
**5**	-	-	-	-	-	-	-	-	-	-	Sometimes	-
**6**	-	Often	-	-	-	-	-	-	-	-	-	-

Note. Therapies have been given a random number from 1-6 for anonymity. “-“ = REMOTION therapist material exercise was not used.

2. Open-ended questions answered by therapists and analyzed with thematic analysis

In the intervention group, 26 different therapists gave written input to four open-ended questions for 24 therapies at T1 and 24 therapies at T2. Written input was analyzed for all text provided at both timepoints using a reflexive thematic analysis approach specified by Braun and Clarke (2022) to answer the following question: *how did REMOTION impact face-to-face therapy*? Author MK coded the data from the four open-ended questions using MAXQDA (Verbi, 2018) and generated initial codes and themes. This was achieved by following the six different phases of thematic analysis described by Braun and Clarke (2022): familiarizing oneself with the dataset, coding, generating initial themes, developing and reviewing themes, refining defining and naming themes and then writing up the results. During theme refinement, themes were also discussed collaboratively with author LLB. The following open-ended items were presented to therapists and their written input analyzed with thematic analysis:

- *If REMOTION was a topic in face-to-face psychotherapy, what was talked about?*
- *Did REMOTION change the therapeutic work with your patient? If so, how?*
- *Did REMOTION have positive effects on face-to-face psychotherapy? If so, what kind?*

- *Did REMOTION have negative effects on face-to-face psychotherapy? If so, what kind?*

The following four main themes were developed during the analysis. Summaries of theme content and exemplary quotes that were translated from German to English are provided to describe the individual themes in the following text:

1. *REMOTION changes the structure and content of face-to-face therapy*

Theme one illustrates that REMOTION had an influence on face-to-face therapy. Patients and therapists were able to discuss specific REMOTION content in therapy, such as exercises from the program. Some therapists also asked their patients about their experience and progress working with REMOTION. One therapist for example mentioned that it was discussed “how the patient is coping with the study, which elements the patient has implemented, and which elements could be integrated into the psychotherapy sessions” (T35). Further, REMOTION allowed certain elements to be outsourced so that it freed up space in face-to-face therapy for other content. REMOTION also supported the therapeutic work regarding emotion regulation. For instance, a therapist described that “the patient asked specific questions about emotion regulation, it was possible to outsource basics” (T06).

2. *REMOTION has positive effects for patients*

Theme two shows that therapists found that patients could benefit from REMOTION in different ways. Some patients mentioned that the general focus of REMOTION was helpful. Patients were also able to improve their knowledge about emotions and emotion regulation as well as their emotion regulation skills. Additionally, REMOTION led patients to work more independently outside of face-to-face therapy sessions. For example, a therapist reported that “it was very helpful, that the patient was able to work on skills independently” (T08).

3. *REMOTION contains negative aspects that may want to be adjusted*

Theme three points out that REMOTION also came with negative aspects. According to therapists, the increased workload was perceived negatively by some patients. A therapist for instance mentioned that “sometimes it seemed that the patient was stressed by psychotherapy AND the program” (T16). A few patients also had difficulties in the implementation of certain exercises. As reported by another therapist “partially, the patient was overwhelmed by the exercises, especially by the mindfulness exercise” (T07). Another negative aspect mentioned was that therapists had too little insight into their patients’ progress, mainly due to not having access to the program.

4. *REMOTION does not always have an impact.*

Theme five accounts for the fact that REMOTION did not have an impact in every case. Some therapists and patients did not use REMOTION. Several therapists also mentioned that there was no influence of REMOTION on the therapeutic work and that REMOTION was not included in conversations between therapists and patients in the face-to-face therapy. For example, a therapist described that “unfortunately, the program could not be sufficiently embedded into the therapeutic process yet” (T12).

3. Therapists' perceived effect on emotion regulation parameters

Table B2. Patient emotion regulation as perceived by the therapists

	**Baseline**		**T1 (observed)**		**T1 (estimated)**		**T2 (observed)**		**T2 (estimated)**		**Group-by-time interaction**	**T1 within-group effect sizes (estimated means)**	**T2 within-group effect sizes (estimated means)**	**Between-group effect sizes at T1 (estimated means)**	**Between-group effect sizes at T2 (estimated means)**
**Measure**	Mean (*SD*)	*n*	Mean (*SD*)	*n*	Mean (*SE*)	*n*	Mean (*SD*)	*n*	Mean (*SE*)	*n*	*F*, *df*	Cohen’s *d* (95% CI)	Cohen’s *d* (95% CI)	Cohen’s *d* (95% CI)	Cohen’s *d* (95% CI)
$DERS_T$															
Treatment	115.65 (19.26)	31	104.24 (18.32)	25	101.31 (3.65)	31	94.08 (18.76)	23	93.62 (3.82)	31	$F_{2, 96.65}$ = 1.40, *p* = .25	0.76 (0.25 to 1.28)	1.16 (0.62 to 1.70)	0.31 (-0.19 to 0.81)	0.42 (-0.08 to 0.92)
Control	115.65 (19.64)	32	106.73 (19.74)	26	107.22 (3.59)	32	102.38 (21.17)	23	102.06 (3.81)	32		0.43 (-0.07 to 0.92)	0.67 (0.16 to 1.17)		
SEK-27-$_T$															
Treatment	47.84 (14.80)	31	53.04 (11.97)	25	55.41 (2.71)	31	62.72 (14.99)	23	63.45 (2.84)	31	$F_{2,95.75}$ = 0.86, *p* = .43	-0.56 (-1.07 to -0.06)	-1.05 (-1.58 to -0.52)	0.06 (-0.44 to 0.55)	-0.27 (-0.77 to 0.23)
Control	47.07 (13.69)	32	56.77 (14.43)	26	56.19 (2.67)	32	58.52 (14.94)	23	59.42 (2.84)	32		-0.65 (-1.15 to -0.15)	-0.86 (-1.37 to -0.35)		

Note. Only therapies for which therapists provided a minimum of T0 (baseline) assessments of the $DERS_T$ and SEK-27_T were included in the analysis. $DERS_T$ = modified version of the Difficulties in Emotion Regulation Scale (DERS, German version, Ehring et al., 2008). SEK-27_T = modified version of the Emotion Regulation Skills Questionnaire (SEK-27, Berking & Znoj, 2008).

References for Supplementary Material B

Braun, V. & Clarke, V. (2022). *Thematic Analysis: A Practical Guide*. Sage.

Berking, M., & Znoj, H. (2008). Development and validation of a self-report measure for the assessment of emotion regulation skills (SEK-27). *Zeitschrift für Psychiatrie, Psychologie und Psychotherapie, 56*(2), 141–153. https://doi.org/10.1024/1661-4747.56.2.141

Ehring, T., Fischer, S., Schnülle, J., Bösterling, A., & Tuschen-Caffier, B. (2008). Characteristics of emotion regulation in recovered depressed versus never

depressed individuals. *Personality and Individual Differences, 44*(7), 1574–1584. https://doi.org/10.1016/j.paid.2008.01.013

VERBI, 2018. MAXQDA 2018 [Computer Software]. VERBI Software GmbH.
